# Editorial: Common tune, different players: emerging molecular guiding factors in development and activity dependent remodelling of different neural circuits

**DOI:** 10.3389/fncir.2023.1221634

**Published:** 2023-06-08

**Authors:** Nicoletta Berardi, Claudia Lodovichi, Paola Tognini, Gabriele Sansevero

**Affiliations:** ^1^Department of Neuroscience, Psychology, Drug Research and Child Health (NEUROFARBA), University of Florence, Florence, Italy; ^2^Institute of Neuroscience, Consiglio Nazionale delle Ricerche (CNR), Padua, Italy; ^3^Veneto Institute of Molecular Medicine (VIMM), Padua, Italy; ^4^Padova Neuroscience Center, University of Padova, Padua, Italy; ^5^Department of Translational Research and New Technologies in Medicine and Surgery, University of Pisa, Pisa, Italy; ^6^Institute of Neuroscience, Consiglio Nazionale delle Ricerche (CNR), Pisa, Italy

**Keywords:** cerebral cortex, plasticity, critical period, circadian rhythm, pupillometry

We wish to dedicate this Research topic to our dear colleague and friend Matteo Caleo, who gave so many important contributions to the study of different forms of neural plasticity. We maintain a vivid memory of him discussing plasticity mechanisms with bright and smiling eyes.

During development, the brain undergoes critical periods characterized by heightened plasticity which involves the remodeling of neural circuits through structural and functional changes in response to environmental stimuli (Takesian and Hensch, 2013). During those sensitive time-windows the structure and strength of synapses are reshaped to reach the final circuit organization which optimizes perception and cognition. At a certain level, activity dependent remodeling of neural circuits takes place throughout life, contributing to experience dependent changes in our brain and behavior (Chakraborty et al., 2021).

In this article collection the complex mechanisms underlying neuroplasticity have been tackled through different perspectives, from molecular to functional point of views, during different life periods, highlighting also underexplored players (such as circadian rhythms or heterosynaptic plasticity).

While most developmental neural changes are thought to involve input -specific homosynaptic forms of experience-dependent plasticity, heterosynaptic plasticity, induced by changes in neighboring synapses, is also an important mechanism often overshadowed by homeostatic plasticity. Our understanding of heterosynaptic (H) plasticity has greatly benefited from new tools and technologies that allow single synapse imaging and manipulation of structure, function, and protein dynamics in living neurons. However, a systematic analysis of in vivo heterosynaptic plasticity phenomena is still particularly challenging and requires a careful and thoughtful approach, to avoid losing heterosynaptic changes within the homeostatic ones. Jenks et al. deeply explore heterosynaptic plasticity and advance novel hypotheses regarding its role in visual cortex plasticity. They propose two mechanisms which contribute to the depression of deprived eye inputs and potentiation of open eye inputs following monocular deprivation. The first mechanism, leading to weakening of synapses from the deprived eye, involves the classical spike-timing-dependent homosynaptic LTD but also compensatory H-LTD (possibly driven by callosal inputs from the open eye). The second mechanism involves facilitatory and cooperative heterosynaptic potentiation, which could be guided by the homeostatic strengthening of existing open eye inputs, promoting the stabilization of new or neighboring open eye inputs. Future research needs to be done in vivo at the synaptic level to determine if and how these heterosynaptic mechanisms contribute to the early and late phases of ocular dominance plasticity by comparing structural plasticity between neighboring and distant synapses, and relating the plasticity to the visual activity of dendritic spines.

The remarkable complexity of brain plasticity is exemplified by the diverse range of signaling molecules that regulate onset and closure of critical periods (CP) (Gibel-Russo et al.). These factors can originate from local neurons and glial cells, as well as extracortical sources (i.e., the choroid plexus), and can involve modulation of the extracellular matrix, myelination and hormonal signaling. Russo et al. review the role of different factors in regulating CP onset, such as BDNF, SPARCL1, and OTX2, and factors which modulate CP closure, such as extracellular matrix, and myelination. Circadian rhythms, hormone, oxidative stress and immune response are also examined, to highlight their impact on CP regulation.

Despite the extensive research on brain plasticity, the role of the circadian clock in experience-dependent plasticity has been largely overlooked. The endogenous clock plays a crucial role in neuronal development, function, and decline in aging (Van Drunen and Eckel-Mahan). Cognitive performance is affected by the time of the day, and several reports show that long-term potentiation, a correlate of memory and learning, is modulated through the day-night cycle although the direction of the effect varies in different studies. While the suprachiasmatic nucleus clock has been deeply investigated for the regulation of daily rhythmicity in physiology, little is known regarding diurnal oscillations in brain plasticity. The possibility that the molecular core-clock regulates cell function through the circadian modulation of mTOR, a master controller of protein synthesis, is analyzed by Lodovichi and Ratto. As mTOR has been involved in synaptic plasticity, the bidirectional relationship between mTOR pathway and the core-clock could be the missing link to explain how the clock might influence brain plasticity. Intriguingly, circadian activation of mTOR seems to be coupled to ion homeostasis, revealing daily cycling of intracellular ion concentrations (i.e., Na+, K+, Cl− and Mg2+). Although changes in ion concentrations along the diurnal cycles have not been demonstrated yet for neurons, shedding light on those mechanisms could have significant implications for neuroplasticity, and chronotherapy in neuropsychiatric disorders.

Finally, the editorial discusses a technique which could open non invasive avenues for the study of cortical plasticity in humans: pupillometry (Viglione et al.). Pupillometry is a promising technique for estimating residual plasticity as pupil dilations coincide with changes in neuromodulatory signaling. In particular, the locus coeruleus is considered a key regulator of cognitive control of pupil size, such that changes in pupil size reflect release of Norepinephrine (NE). Studies have shown that monocular deprivation affects spontaneous slow pupil oscillations and that more prominent pupillary fluctuations are associated with robust alterations in ocular dominance during binocular rivalry dynamics. Pupil size has also been used to study switches between alternative percepts, with previous findings suggesting a transient dilation during perceptual switching indicating an increase in norepinephrine levels. Recent studies have deconstructed the complex pupillary response during changes in perception, showing that the amplitude of constriction but not dilation is systematically modulated by the duration between perceptual changes. Norepinephrine plays a role in regulating pupil diameter modulation and visual cortical plasticity, and the close association between norepinephrine tone and pupil diameter demonstrates the potential of pupillometry as a valuable tool to study adult cortical plasticity in clinical populations.

As we continue to explore the intricate mechanisms underlying neuroplasticity, we are discovering new and exciting ways to study the brain and rescue its physiological function. Recent advances in technology and techniques, such as single synapse imaging and pupillometry, are shedding light on the complex processes involved in neural circuit remodeling during critical periods of development and throughout adulthood. Short and long range interactions seem to cooperate to finally shape neuronal circuits through specific activation of biochemical cascades and transcriptional programmes, and a comprehensive understanding of how these signals interplay and are regulated by circadian rhythms might help to provide insight into the effects of early adversity and developmental defects on perception and behavior.

The future of neuroplasticity research is bright, and with the continued development of innovative techniques and approaches, we are sure to conquer even more precise insights into the brain's ability to adapt and learn throughout our lives and to understand how to harness the different forms of neuroplasticity to promote adaptive responses to environmental stimulations and favor brain repair.

## Author contributions

This Research Topic was established through unanimous agreement among all the editors. They worked collaboratively to determine the acceptance or rejection of papers, and each manuscript underwent review by both the panel of editors and peer reviewers. Every member of the editorial team contributed their insights and revisions to shape the final published document.
